# Modelling a Silent Epidemic: A Review of the In Vitro Models of Latent Tuberculosis

**DOI:** 10.3390/pathogens7040088

**Published:** 2018-11-15

**Authors:** Savannah E.R. Gibson, James Harrison, Jonathan A.G. Cox

**Affiliations:** School of Life and Health Sciences, Aston University, Aston Triangle, Birmingham B4 7ET, UK; gibsoser@aston.ac.uk (S.E.R.G.); j.harrison11@aston.ac.uk (J.H.)

**Keywords:** tuberculosis, latency, non-replicating persistent, antibiotic, drug discovery, *Mycobacterium tuberculosis*

## Abstract

Tuberculosis (TB) is the primary cause of death by a single infectious agent; responsible for around two million deaths in 2016. A major virulence factor of TB is the ability to enter a latent or Non-Replicating Persistent (NRP) state which is presumed untreatable. Approximately 1.7 billion people are latently infected with TB and on reactivation many of these infections are drug resistant. As the current treatment is ineffective and diagnosis remains poor, millions of people have the potential to reactivate into active TB disease. The immune system seeks to control the TB infection by containing the bacteria in a granuloma, where it is exposed to stressful anaerobic and nutrient deprived conditions. It is thought to be these environmental conditions that trigger the NRP state. A number of in vitro models have been developed that mimic conditions within the granuloma to a lesser or greater extent. These different models have all been utilised for the research of different characteristics of NRP *Mycobacterium tuberculosis*, however their disparity in approach and physiological relevance often results in inconsistencies and a lack of consensus between studies. This review provides a summation of the different NRP models and a critical analysis of their respective advantages and disadvantages relating to their physiological relevance.

## 1. Introduction

Tuberculosis (TB) is the ninth leading cause of death in the world and is the primary cause of mortality by a single infectious agent [1]. According to the World Health Organisation (WHO) there are more than 10 million new cases of TB recorded every year; particular hotspots for TB incidence include Sub-Saharan Africa and South-East Asia. There were an estimated 1.7 million fatalities caused by TB in 2016 of which 375,000 were in HIV-positive people who bear a heavy burden of TB disease, however the global TB mortality rate is falling at 3% a year [1]. The causative agent of TB, *Mycobacterium tuberculosis*, is spread by aerosolisation when infected individuals cough. The exhaled droplet nuclei carry *M. tuberculosis* which is then inhaled by a nearby individual [2]. The infectious dose for *M. tuberculosis* infection is around 1–5 bacilli [3]. *M. tuberculosis* progresses to the lungs, where they largely inhabit the resident professional phagocytes. As the disease progresses, neutrophils, monocytes, and eventually dendritic cells are recruited by distress signals from the infected macrophages [4,5]. These innate immune cells are then infected as well, compounding the problem. When the adaptive immune system takes control, the bacteria mainly arrest their growth and symptoms become transient or non-existent [6]. In immunocompetent individuals, the adaptive immune system is able to contain *M. tuberculosis* infection by sealing the bacteria in a cooperative group of cells from the innate and adaptive immune system that isolate the bacteria from the rest of the body [7,8,9]. At this point, progression to latent disease occurs in up to 90% of individuals. If the granuloma cannot be maintained due to immune impairment, *M. tuberculosis* is released and the infection progresses to active TB disease. At this point the individual becomes infectious and starts to shed bacteria [10]. They also become symptomatic: general symptoms of TB include fatigue, weight loss, and coughing up bloody sputum [11]. Growing incidences of drug resistance, a high burden of disease and increasing socio-economic determinants such as war and high levels of poverty indicate that more action is needed to eradicate this expanding public health problem [12,13,14,15].

To achieve the WHO “End TB Strategy” objective of a 90% reduction in TB by 2035, a unified strategy which improves diagnosis and treatment of both latent and active TB is crucial [14,16]. Latent TB, otherwise known as Non-Replicating Persistent (NRP) TB [17], is one of the main mechanisms of TB virulence. It can survive in the host for decades without becoming symptomatic and will only reactivate when the host becomes immunocompromised, even to a small degree [10,18,19]. Latent TB is not a faithful term for the changes that *M. tuberculosis* undergoes. The term “latent” often refers to a dormant state with no active metabolic processes and no response to environmental stimulus. This is not true in the case of TB: its metabolism is regulated to an essential level but is still functional [20,21,22]. The phrase Non-Replicating Persistence (NRP) was first used by Wayne in 1976 and has since become adopted as the appropriate term to describe this state [17,23].

Latent TB is diagnosed by a positive Tuberculin Skin Test (TST)—which produces an antigen (tuberculin) specific T cell response—without the presence of symptoms [24,25]. By mathematical modelling, it has been estimated that 1.7 billion people are latently infected with TB [26]. Of these, around 56 million are deemed highly likely to reactivate into active disease [26]. It is highly likely that a large proportion of the latent disease is drug-resistant and so could reactivate into MDR TB [19]. Both forms of the disease are exceptionally hard to treat and even with intensive combination therapies, treatment is only 54% successful [1]. The lack of effective treatment options for MDR TB is a problem that will only increase with the spread of antibiotic resistance [27]. Therefore, a treatment that effectively targets the asymptomatic, latent state of TB is preferable to current therapies; this would also help to eradicate the currently daunting reservoir of active infection as 90% of all infections are latent [1,14,18]. 

When *M. tuberculosis* is contained by the adaptive immune system within the TB granuloma [28], a distinct metabolic and physiological shift takes place [21,29]. The genes expressed are distinctly different to the active phenotype [29]. This genetic shift is now thought to be caused or largely influenced by the metabolism of cholesterol [21], instead of other preferable fatty acids (glycerol) or glucose [20]. Cholesterol is known to be the only carbon source present in the granuloma as, over time, all other carbon sources have been used by the bacteria whilst still active [20,30]. 

Treatment of TB is preferable when the disease is in the NRP state and the patient is not expressing any symptoms. To do this, novel compounds require screening against an in vitro model of NRP TB. There have been a few different models of NRP TB developed all with unique advantages and disadvantages. These different models can all be utilised for the research of different physiological characteristics of the NRP state. This review provides a summation of the different NRP models and a critical analysis of their respective advantages and disadvantages. 

## 2. Conditions within the Granuloma

**Figure 1:**
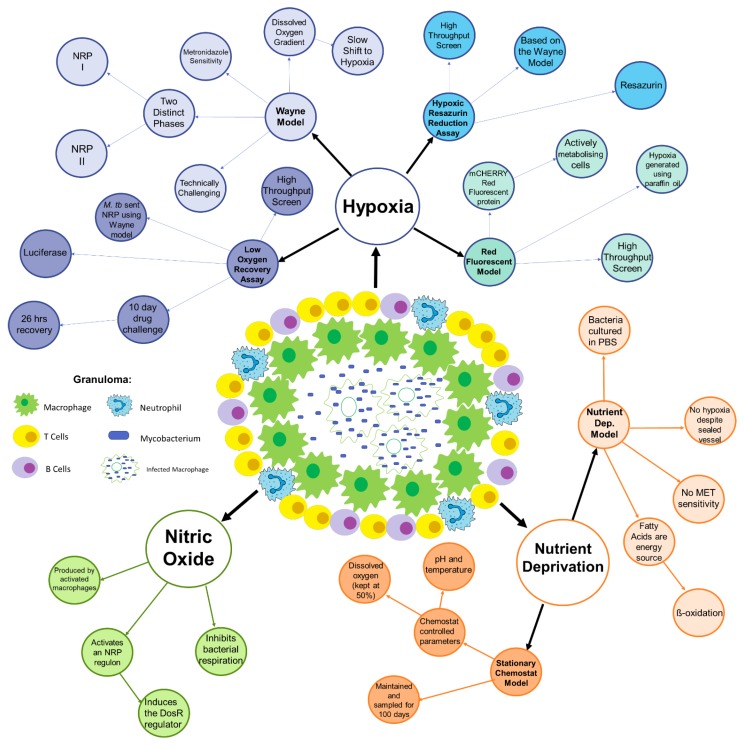
A summary diagram of the in vitro models of Non-Replicating Persistent (NRP) Tuberculosis categorised by the granuloma condition it models.

Conditions found within the granuloma are key to the NRP state and accurately mimicking these conditions in vitro allows for the development of new models. The environment in the granuloma has a distinct profile that includes hypoxia [17,23], nutrient deprivation [31,32,33], limited carbon sources [21,22,33], and a high concentration of nitric oxide (NO) [34]. Most of the above environmental conditions have been shown to induce the NRP state in mycobacteria individually. It could be presumed that the combination of all these conditions will produce a phenotype closest to that found clinically. Nevertheless, most in vitro models focus on one of the conditions in isolation—although there are a few that combine two conditions in their model. A summary of the models discussed can be found in Figure 1. 

## 3. Hypoxia

Hypoxia and the gradual depletion of oxygen is a key element of the granuloma [9]. Upon detection of an oxygen gradient, *M. tuberculosis* starts to prepare for the NRP state [17,23]. Hypoxia was one of the first conditions of granuloma identified and as such, it is the best characterised. The following models all focus on modelling the hypoxic element of the granuloma to trigger the NRP state, starting with the original and most famous NRP model, the Wayne Model [35].

### 3.1. The Wayne Model

In 1976, Lawrence Wayne made the observation that whilst an *M. tuberculosis* culture was aerated, growth would continue in a logarithmic fashion; if aeration was stopped, the culture settled and the concentration of dissolved O_2_ (dO_2_) decreases, growth would arrest seemingly indefinitely [23]. The concentration of dO_2_ was increased by shaking, which lead to the continuation of exponential growth after an extended period of time in an arrested state. This discovery of the effect of an oxygen gradient on *M. tuberculosis* was the first indication that *M. tuberculosis* could enter a state similar to latency, but being subtly different. He coined the state Non-Replicating Persistence (NRP) to reflect the differences [17,35]. After a few improvements, Wayne introduced an in vitro model of Latent TB based on his observations of the effect of hypoxia. His hypoxic model, termed The Wayne Model, was introduced in 1996 [35]. The aim of this model was to simulate the gradual depletion of oxygen in the granuloma. The organisms were grown in sealed containers with a controlled ratio of air to culture medium equalling 0.5. This ratio is called the Head Space Ratio (HSR). As the culture grows aerobically, it slowly uses up all the oxygen in the HSR: thus, creating the slow shift down into anaerobic conditions due to the reduction in dO_2_. This model contains two distinct states of NRP. The first occurs just as the oxygen saturation in the HSR reaches 1%. Wayne called this NRP stage I [17,35]. This stage is described as “microaerophilic”, where the bacilli are no longer replicating or conducting DNA synthesis but still have high levels of ATP production and some active mechanisms of DNA repair [17,29,36]. This is followed by NRP stage II, characterised by fully anaerobic conditions defined as below 0.06% oxygen saturation [35]. NRP stage II is the phenotype most often referred to when describing NRP *M. tuberculosis.* It is important to note that *M. tuberculosis* cannot survive if placed straight into NRP stage II conditions: the process of steady decrease in oxygen saturation in NRP stage I is necessary to achieve NRP stage II [35]. Hypoxia is confirmed by the decolourisation of methylene blue (concentration of 1.5 µg/mL) and by a stabilisation of the growth curve into a plateau [37], sometimes referred to as an early stationary phase. Under this model, *M. tuberculosis* is indifferent to the presence of Isoniazid (INH) but the presence of Metronidazole (MET) has a bactericidal effect [38]. This is directly opposed to the effect of these drugs in aerobic conditions where INH has a bactericidal effect on *M. tuberculosis* but MET has no inhibitory effect [39]. 

This model is the first to model in vitro NRP *M. tuberculosis*, and is still the model of choice for most Latent TB researchers. Whilst this model has facilitated a great increase of knowledge into Latent TB and its metabolic profile, it does have some limitations. Firstly, the bactericidal effect MET has anaerobically is not reflected in animal models, such as the Cornell mouse model [40] and a guinea pig model [41]. This has led to the assumption that MET would have no effect if used therapeutically and has cast doubt on other active compounds identified using the Wayne model. 

This is perhaps related to the Wayne model singularly focussing on replicating the slow shift to hypoxic conditions that happen in the granuloma; it does not include any other environmental conditions found in granuloma [42] (Figure 1). These other factors have an effect on the physiological and metabolic profile of the *M. tuberculosis* which would cause the bacteria to react in a different manner to challenges. Therefore, as the Wayne model lacks these other physiologically relevant conditions, any NRP active antimicrobials identified using this model are treated with some speculation. The bacteria have a different physiological and metabolic profile in vivo and this is reflected in the difference in drug profiles [41].

Nevertheless, this model is still frequently used in research and has provided large contributions of knowledge and insight into NRP physiology. In addition, a large majority of recent models borrow heavily from the Wayne model. Therefore, this primitive starting point has paved the way for a multitude of other models for NRP in *M. tuberculosis*. 

### 3.2. Hypoxic Resazurin Reduction Assay (HyRRA)

The following model is an example of an in vitro model that has a focus on high throughput phenotypic screening (HTPS). With the demand for new antimicrobials ever increasing, HTPS has become the method of choice for identifying novel active antimicrobials [43]. Whilst HTPS commonly lacks specificity compared to other testing methods, the ability to quickly and frugally screen high volumes of novel compounds to identify new inhibitory molecules is both cost and time efficient. 

The HyRRA model is based on principles from the Wayne model [35] and an aerobic HTPS *M. tuberculosis* assay called Resazurin Microtitre Assay (REMA) [44]. Colorimetric assays such as REMA or an Alamar blue assay have become as common as rapid, inexpensive methods of visual minimum inhibitory concentration (MIC) identification [44,45].

The HyRRA was tested on *M. tuberculosis* H37Rv, *Mycobacterium smegmatis*, and *Mycobacterium bovis* BCG. All species were cultured in 3 mL aliquots in sealed vacutainer tubes, then kept static to induce hypoxia. Drugs were then aseptically added, and the dosed cultures were incubated for 96 h. After this point, the cultures were dispensed into microtitre plates and 0.02% resazurin was added. Resazurin is reduced to Resorufin in the presence of metabolically active cells, thus causing a colour change from deep purple to pink [46]. This cell viability assay was then used to screen a large antibiotic panel using this model, and compare the MICs of these compounds against previous models’ findings and classic colony forming units (CFU) assay [47]. The MICs identified by the colourimetric assay were found to be comparable to those found from CFU counts. They found activity against NRP TB from compounds from the nitrofuran group [48]. In this model, as with the Wayne model, the bacteria tested show sensitivity to MET, potentially due to the shared hypoxic condition. 

This model facilitates the down-scaling of NRP *M. tuberculosis* drug testing to enable a HTPS, improving the discovery of new antimicrobials expeditiously. This is a considerable advantage as previous models struggled to adapt to screening a large quantity of novel compounds. As with the Wayne model, the HyRRA model is based on the hypoxic environment found inside the granuloma. The presumption made is that if hypoxia alone can trigger entry to the NRP state, then hypoxia alone is enough to model the granuloma [17,35]. This is partially correct: hypoxia does trigger entry into the NRP state and will maintain the bacteria in this state, so it is correct to presume that hypoxia is a large driving factor of NRP. However, as discussed later in this review, hypoxia is not the only stress condition present in the granuloma with the ability to trigger the NRP state (Figure 1). The HyRRA solely focusses on one stress condition that can induce the NRP state in mycobacteria. This induction facilitates compound testing on mycobacteria in the NRP but without the other conditions, the compound testing will never be physiologically relevant and as such will produce many false positives. Additionally, many compounds could take longer than 96 h to depict a sterilising action and so this method could exclude some potential compounds.

### 3.3. Low Oxygen Recovery Assay (LORA)

Another model which is more adapted to HTS is the Low Oxygen Recovery Assay (LORA) [49]. Large elements of this model are based on the Wayne model [35] and as such could potentially be characterised as an adaptation of the Wayne model instead of a standalone model. The LORA assay makes use of a luciferase reporter (*luxAB* gene) [50] to depict the metabolic activity level of cells and the authors showed that, on entrance to the NRP state, luminescence decreased but remained present and constant as the experiment progressed [49,51]. In short, the recombinant *M. tuberculosis* H37Rv was manipulated into NRP stage II using a similar protocol to Wayne’s [35], albeit using a chemostat to accurately control conditions such as dO_2_. After 22 days under these conditions with regular optical density readings (OD_570nm_), CFU counts, and Relative Light Unit (RLU) readings taken, the cultures were spun down in Phosphate-Buffered Saline (PBS) and frozen at −80 °C. These stocks were challenged with antimicrobial agents for 10 days under anaerobic conditions and then given a day’s aerobic recovery. Again, luminescence and CFU counts were taken. 

To determine the suitability of this assay’s use as a HTPS, a Z’ test was conducted [52]. The LORA’s Z’ factor was determined from the RLUs after 10 days of anaerobic incubation and was determined to be in the range of 0.58–0.84. A Z-factor value between 0.5 and 1 is indicative of an excellent assay that is suitable for HTS, therefore the LORA is suitable as a HTPS [52].

The authors tested 31 antimicrobial compounds using this model and compared this to a comparative aerobic counterpart and previously recorded results. As found in the Wayne model, INH, which targets the cell wall [53], has no effect on NRP *M. tuberculosis* [17,39]. This lack of efficacy is also consistent clinically. Other drugs that have cell wall targets were also found to be inactive such as Ethambutol and Cycloserine [49]. In agreement with previous models finding: MET [38], Capreomycin [54], and Moxifloxacin [55] had strong sterilising activity among some other active compounds. The general conclusion drawn is that cell wall targeting drugs become inactive in NRP. However, those drugs with intracellular targets such as MET and compounds, including Capreomycin that target the 30S ribosomal subunit, gain activity [38,54].

An example of the LORA being used to identified novel compounds was shown by Bonnett et al. where they identify hydrazones as active against NRP *M. tuberculosis* [56]. These hydrazones were previously identified as effective compounds against active TB. The drug target was found to be the enzyme LepB which is a crucial part of the general secretion pathway of TB [57].

The LORA model has many advantages as a model and as previously discussed, the world of drug discovery has an ever increasing focus on HTPS [43]. The LORA’s suitability for HTPS as confirmed by the Z’ [52] is encouraging; as the authors showed, a wide variety of compounds can be screened with comparative ease when compared to the Wayne Model [35,38]. The use of a luciferase reporter to monitor entry to the NRP state as well as drug activity is novel. This provides a wider range of information than what could be gleaned from previous models such as the HyRRA which uses a qualitative measure to determine the culture entry to NRP [47,49]. 

Nevertheless, as with all models, there are some disadvantages to using this in vitro model. Similar to the HyRRA and the Wayne model, this model is based exclusively on hypoxia [35,47,58]. As previously discussed, this is an important element but is not independent clinically (Figure 1). 

Secondly, this is a model based on determining the MIC of novel compounds whose activity and target may not have been identified. The luciferase reporter enabled assessment of the metabolic activity observed in the NRP state. However, to transform the *M. tuberculosis*, a kanamycin selective marker was used [59]. This means that the recombinant *M. tuberculosis* H37Rv-*luxAB* is resistant to kanamycin. This has the potential to confer some level of resistance to other antimicrobials. This is especially relevant when conducting a HTPS on novel compounds. This could lead to the elimination of some compounds that clinically could have powerful sterilising activity. 

Finally, this assay requires special instruments (Anoxomat system) and is expensive to run with a high cost of reagents and equipment. Generally, the optimal HTPS should be as inexpensive as possible because of the potential low yield of active compounds [43]. 

### 3.4. Red Fluorescent Protein (RFP) Model

This model is also a HTPS of NRP *M. tuberculosis* that is based on the hypoxic element of the granuloma [60]. This model exposed the disadvantage of previous hypoxic models [35,49] which was to maintain hypoxia, all elements of the experiment (both culture and compound) are added together and sealed or placed in an anaerobic cabinet. However, entry to the NRP state takes a period of time extending from 48 h to 120 h dependent on conditions [23,31,35]. For approximately the first 72 h in previous models, the *M. tuberculosis* was still in its active state. Therefore, as some compounds (for example rifampicin) are very fast acting compounds; activity to NRP *M. tuberculosis* could be shown. In fact, the compound would have been faster to sterilise the culture than the *M. tuberculosis* was to turn NRP. The Red Fluorescent Protein (RFP) model aims to overcome this hurdle by combining molecular biology techniques and a different method of excluding oxygen.

Red fluorescent protein can be utilised as a reporter for gene expression and so can be used to determine the difference between an actively growing culture, a static culture and a culture affected by a bactericidal drug [61]. RFP protein was transformed into *M. tuberculosis* H37Rv using the pCHERRY3 plasmid [62].

This model also made use of microtitre plates to conduct a HTPS. Cultures were grown aerobically and then a layer of paraffin oil was added on top of the culture which oxygen cannot permeate [63]. To test if the culture is hypoxic, methylene blue was added (1.5 µg/mL), which decolourises in the absence of oxygen [35]. This was incubated for 13 days, at which point compounds were injected into the hypoxic cultures through the paraffin oil layer. This was then incubated for a further 20 days with daily fluorescence readings taken [60].

A wide range of compounds were tested, each chosen for their differing modes of action [60]. A notable feature of all the previous hypoxia models is sensitivity to MET [35,47,49]. Interestingly, the RFP model does not show any sensitivity to MET. The authors postulate that this discrepancy could be due to MET being a pro-drug and its activation is largely based on the state of the bacilli [60]. However, this sensitivity to MET is not seen in any in vivo test, therefore, this lack of sensitivity could indicate an improved physiological advantage to the model [41]. This model also highlighted the extended period of time needed for some compounds to show activity such as the aminoglycosides. Some previous models did not expose the cultures to the compounds for this extended period of time. The drug resistant nature of the bacilli can require a large lead time before the compounds take effect [64].

As in the previous tests, a Z’ analysis was conducted to see whether this model is suitable for a HTPS [52] which gave a value between 0.91–0.94, indicating that this model is robust for HTPS. 

The main advantages of this model have already been touched upon. Briefly, other models previously exposed cultures to compounds before they had gone fully into the NRP state [31,35,47,65]. This model ensures that compounds are only tested against *Mycobacterium* that have fully entered the NRP state. Secondly, this model shows that the bacteria are demonstrably in the NRP state, however, there is no susceptibility to MET. Therefore, it could be postulated that hits generated using this model are more physiologically relevant than those identified by previous models. Finally, this model exposes the NRP cultures to compounds for an extended period of time compared with previous models. Some compounds take a long time to act on this highly resistant phenotype of *M. tuberculosis*; so a shorter period of time could exclude some compounds that have a high efficacy but need longer to take effect.

Introduced in 2018, this model represents the most recent offering towards NRP research and addresses some of the issues with previous models. Nevertheless, no model perfectly simulates the clinical, in vivo condition and there are disadvantages associated with this model. As with the LORA, this model utilises a transformed version of *M. tuberculosis* [49]. This involved the transformation of *M. tuberculosis* H37Rv with RFP using the pCHERRY3 plasmid, which uses a hygromycin selective marker [61]. Using a culture that already has some antimicrobial resistance is not ideal as it could lead to some level of cross resistance to other antibiotics. 

In addition, hypoxia is the only element of the granuloma being imitated in this model, as in the other models. As the subsequent models will demonstrate, other NRP-inducing conditions have similar but not identical transcriptomes [66]. To create a model that is physiologically relevant, all conditions should be taken into account (Figure 1). 

## 4. Nutrient Deprivation and Selective Carbon Sources

**Figure 2:**
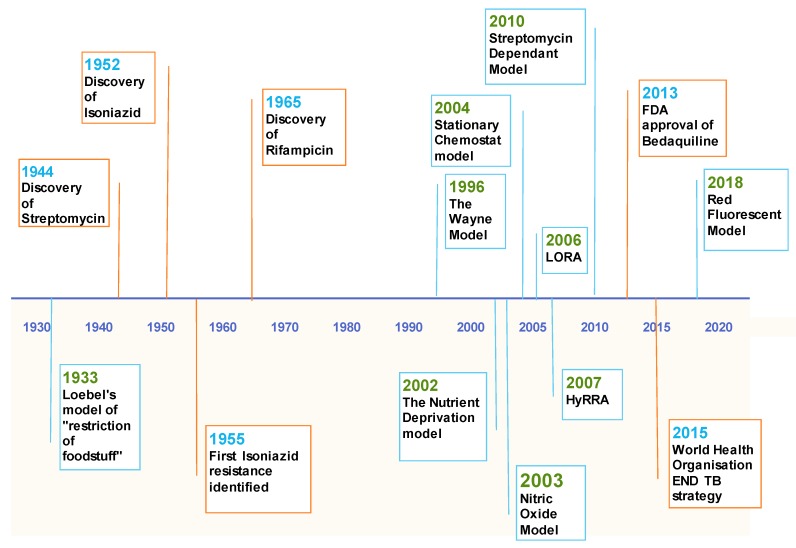
Timeline of in vitro NRP models.

As early as 1933 (Figure 2), nutrient deprivation was indicated as able to induce the NRP state in TB [32,67]. In recent years, this work has been further developed and has shown granuloma-based bacteria that are not only nutrient starved [31,68], they are restricted to odd chain fatty acids as the sole carbon source, namely cholesterol [21,22]. The effect of nutrient starvation has been less studied than hypoxia; however, the following models all show that they can model NRP *M. tuberculosis* albeit with a different drug sensitivity profile to that observed in hypoxia-derived NRP mycobacteria. 

Figure 2 presents a representation of the introduction of in vitro models of Non-Replicating Persistent Tuberculosis in combination with the landmarks of Tuberculosis research. Orange markers represent milestones in TB discovery and treatment. The blue markers represent the introduction of the varying models, where LORA indicates the Low Oxygen Recovery Assay and HyRRA is an abbreviation for the Hypoxic Resazurin Reduction Assay.

### 4.1. The Nutrient Deprivation Model

In 1933, in vitro TB research was still relatively new; Loebel and his team demonstrated that it is possible to transfer an *M. tuberculosis* culture out of rich media into PBS [32], which then can be left in solution for many years (Figure 2). Respiration levels slowly decreased and the culture remained in early stationary phase; however, upon reintroduction to rich media, respiration levels increased and the bacterial cells resumed normal growth [67]. Loebel concluded that it was possible for *M. tuberculosis* to survive for an extended period of time and that this virulence factor could be attributed to the bacteria’s ability to “depress its oxygen consumption and to live off previously stored foodstuffs”. This postulate was later proved to be correct by subsequent models [17,31]. 

*M. tuberculosis* from a granuloma has a different morphology to those grown in vitro, however, nutrient starved *M. tuberculosis* has a similar morphology to the in vivo phenotype [33]. This would suggest that nutrient starvation is an essential environmental condition in the granuloma with an altered genetic profile that in vivo could work in conjunction with hypoxia activated genes to produce the clinical phenotype [20,58,69]. Betts and her research team came up with a model based on Loebel’s earlier work that would stop respiration and halt replication but keep the bacteria viable [31,32,67]. 

In this model, bacteria are grown for 7 days in nutrient rich media at which point they are pelleted and resuspended in PBS. They are incubated at 37 °C in sealed containers [31]. Viability is determined by CFU counts at sequential points. Despite no growth at any point, the CFU counts remained consistent throughout, which indicated that the NRP state had been achieved. Interestingly, despite being cultured in a sealed container, similar to the Wayne model, there is no decolourisation of methylene blue which shows that oxygen is still present in the cultures [31,42].

The Wayne model was used as a control and as previously seen, after 10 days in sealed containers containing rich media, the culture decolourised methylene blue and entered hypoxia [35]. 

This led to the hypothesis that, instead of the oxygen being consumed, as in the Wayne model [35], the bacilli slowed down their respiration levels and thus entered the NRP state. In this model of NRP, bacteria gain resistance to INH and RIF, however, they do not gain susceptibility to MET [31]. This is one of the primary differences between the Nutrient Deprivation model and the Wayne model [17,31]. They also noticed a difference in gene expression in response to nutrient starvation. They found many enzymes concerned with energy metabolism are downregulated under nutrient-deprived conditions. These enzymes included ones in the tricarboxylic acid (TCA) cycle (*fum*, *acn*, *icd1*) and in glycolysis (*gap*, *tpi*). Sigma factor B (*sig*) was also found to be upregulated. Expression of *sigB* has been associated with the transition into stationary phase and has also been associated with stress conditions [70,71]. An analysis of the whole transcriptome of *M. tuberculosis* in both models showed many similarities including an adaptation in metabolism. However, whilst the model shared 50 “top scoring” genes with the Wayne model, there were over 200 different upregulated genes [66]. 

This is also a widely accepted model of NRP, made interesting by its different drug susceptibility to the Wayne model. This difference could be attributed to its distinctly different transcriptome [66]. Nevertheless, entry into the NRP state can be observed despite oxygen being abundant [31]. All the above evidence seems to imply that both nutrient starvation and hypoxia are essential conditions in the granuloma to provide the right environment for NRP.

From this, both models have the same failure of only looking at one environmental factor without reflecting the full picture of physiological conditions within the granuloma (Figure 1).

### 4.2. Stationary Chemostat Model

Building on this work into investigating the effect of nutrient deprivation on *M. tuberculosis*, a new model was proposed which aimed to use a chemostat to tightly control conditions such as pH, temperature, and dissolved oxygen [31,32,67,72]. This stationary chemostat model would allow the long-term maintenance of an NRP culture. Chemostats have been utilised by scientists attempting to culture many different bacteria under challenging conditions as it allows greater control of the environment than traditional culture methods [73,74,75].

This model cultured *M. tuberculosis* H37Rv in 750 mL of ADC enriched Middlebrook 7H9 broth with a defined dissolved oxygen concentration of 50%. This culture was then maintained until all the nutrients has been depleted; this slowing of growth was defined as stationary. The depletion of glucose and glycerol was monitored by biochemical assays over the duration of the experiment. Culture samples were extracted from the chemostat at intervals throughout the experiment and plated for CFU counts. To monitor the transcriptome of the culture, RNA was extracted at various time points throughout the experiment. 

The authors have based this model on the theory that there is a proportion of bacteria that go into an extended stationary phase in response to an external pressure, similar to what is seen in *Escherichia coli* [76]. This could be generated in vivo by exposure to antibiotics to which a small proportion of the population would survive (persister population). They observed what they have defined as stationary phase up until day 80, which they attribute to nutrient deprivation, at which point the culture restarts growth. This revival is hypothesised to be the result of adaptation to the new growth environment. 

The main advantage of this model is that it is conducted in a chemostat, which had not previously been explored as an option for NRP *M. tuberculosis.* Rigidly controlling the environment to simulate known conditions in the granuloma is a widely employed method of in vitro modelling. 

The theory that the condition of Latent TB is caused by stationary persisters as discussed by this study requires further validation. This would have merit if the bacteria were solely extracellular and if this phenomenon did not occur in individuals who had not received antibiotic chemotherapy for their TB [10]. An interesting facet of *M. tuberculosis* infection is the ability to survive extracellularly and intracellularly [77]. It has long been thought that the primary infection is driven by the extracellular bacteria; the intracellular bacteria (predominately residing within the macrophages) are the bacteria involved in the granuloma [17]. Hence, it is the intracellular bacteria that are mainly exposed to the conditions of the granuloma which are the driving force to the persistence of *M. tuberculosis* [17,78]. In addition, the culture showed a resuscitation at 80 days; as early as 1933, it was shown that *M. tuberculosis* can persist in sealed containers for 12 years [79]. This evidence in addition to patients reactivating after 20 years provides compelling evidence that this model does not achieve the persistent sate observed clinically [64]. 

Another hallmark of persistence of *M. tuberculosis* is the cessation of replication as observed by previous models [31,39,79]. The growth curves displayed in this model do not show a stable, persistent population but a population in a slow decline [72]. This type of growth curve is more reminiscent of a culture in the decline phase of the growth curve: attributed to the depletion of glucose and glycogen. It is possible that the culture has not entered the NRP state but instead has progressed into decline phase, and before this could complete, the bacilli found a new source of nutrients. A large amount of Tween 80 is used in the medium (0.2%), the stereotypical level of Tween 80 in mycobacteria cultures is 0.05%. It has been identified that mycobacteria can utilise Tween 80 as a carbon source [80,81]. Therefore, whilst the culture has been deprived of glucose and glycerol—which could contribute to the culture’s longevity—it cannot truly be described as nutrient deprived as there are alternative carbon sources present [72]. The original nutrient deprivation model utilised PBS, as have subsequent models, and have demonstrated long term persistence and viability [31,32]. 

This model has many promising features, such as the innovative use of a chemostat for NRP *M. tuberculosis* culture. However, to be utilised as a strict model of NRP, the media used in this study may need to be reviewed to reflect the long term persistence seen in other models [31,39].

## 5. Nitric Oxide

The previous models have highlighted the two best known environmental conditions of the granuloma: hypoxia and nutrient deprivation [17,31]. Nevertheless, there are other lesser studied environmental conditions that can induce *M. tuberculosis* to enter the NRP state, such as the presence of nitric oxide (NO). Activated macrophages produce NO as a signalling molecule and as a potent antibacterial chemical [34]. NO has also been associated with the inhibition of mitochondrial and bacterial respiration [82]. It has also been shown that NO is responsible for the control of mycobacterial replication, along with various other cytokines and chemokines, such as interferon-ɣ and tumour necrosis factor-α [83]. 

This model investigated whether NO would trigger NRP; as a low, non-toxic concentration inhibits bacterial respiration. Inhibited respiration could led to the same state as hypoxia, since hypoxia also limits respiration, but by the depletion of oxygen [84]. *M. tuberculosis* is cultured in the widely used Middlebrook 7H9 broth in aerobic conditions but with a subtoxic concentration of NO. The authors introduced this as less of a structured model of in vitro NRP and more of a study into whether NO can independently trigger the NRP state. 

Exposure of *M. tuberculosis* to NO was shown to induce a 48-gene regulon via the DosR regulator [29]. The DosR regulator or the dormancy survival regulator was identified previously using the Wayne model as being essential for survival in hypoxic conditions [85,86]. DosR is responsible for activating one of the key NRP genes, *acr* (*M. tuberculosis* alpha-crystallin/Rv2031), which has been shown to be essential for the growth of *M. tuberculosis* in macrophages [86,87]. 

NO was also showed to inhibit mycobacterial respiration and halt replication in this model. Evidence that would suggest that NO is key to NRP state is the activation of key genes that seem to show that under hypoxic conditions, nitrate becomes the terminal electron acceptor [88].

The effects of NO on *M. tuberculosis* induces the same genes and thus physiology as hypoxia does, albeit via a very different methodology. The induction of the DosR regulon, the cessation of growth and the inhibition of respiration are all key markers of the NRP state in both hypoxia and nutrient deprivation [82,89]. The ability of NO to independently produce a similar phenotype to both other conditions highlights the importance it must have in the clinical phenotype. 

This has not yet been developed into a functional model and there have been no drug panels tested against it. Nevertheless, as it shares such a close phenotype to that of hypoxia, the presumption is that the drug profile should be, by and large, the same [54]. The discovery that NO can induce the NRP state in *M. tuberculosis* is a leap forward in knowledge concerning this physiological state and its triggers. However, the NO model requires further development before it can be compared with the other models [31,39].

## 6. Streptomycin Dependent 

Finally, there is a Streptomycin-dependent model which utilises the 18b strain of *M. tuberculosis* which has mutated to only grow in the presence of Streptomycin [90]. When this antibiotic is removed, replication ceases [91]. The theory behind this model is that this cessation of replication due to the removal of Streptomycin could mimic the NRP state [17,91]. Cultures were grown in Middlebrook 7H9 media in the presence of 50 µg/mL of Streptomycin. The Streptomycin is removed and the cultures are then starved for two weeks before being exposed to antimicrobial compounds. The protocol for drug testing is the REMA and it is the same method that the HyRRA is based on [44,47].

This model reports an altered drug profile to those seen in more developed models [91]. A full drug panel was screened against the model and showed no activity from INH but an increased susceptibility for front-line antibiotic Rifampicin [91]. Also identified was the strong sterilising action of new TB compound of interest, PA-824 [92,93]

This model attempts to mimic entry into the NRP state via the removal of streptomycin. However, the NRP state is still not fully understood: the transcriptome and physiology can vary between different models that exhibit different granuloma conditions [17,31]. This model was presented as an easy, affordable, and reliable way of conducting a HTPS on NRP mycobacteria. The altered drug profile observed when using this model casts doubt on the ability to accurately screen in vitro for effective drugs in vivo [19,40]. This is coupled with the model not mimicking any part of the granuloma, and as we do not yet know the implications of these environmental conditions, crucial elements of the NRP state could be missing from this model.

## 7. Summary

Despite recent interest, there is still a large void in knowledge concerning the NRP state, both genetically and physiologically (Figure 2). Many attempts have been made at modelling the NRP state in vitro, all contributing different approaches and goals. However, there hasn’t yet been a widely accepted model proposed that mimics more than one aspect of the granuloma. Trying to replicate just one condition has a lot of merit as it allows a deep investigation into the effects of one variable on the bacteria. The other argument is that if just one condition in isolation can trigger the NRP state, combining all the other conditions in one unwieldy model is unnecessary. 

However, when modelling a bacterial infection with the purpose of novel drug screening, the model needs to be as representative of the clinical disease as possible. As the above models show, the different environments all induce a clearly distinct NRP state with different genetic profiles and drug susceptibility (Figure 1). The current practice to address this issue is to use several of the models previously discussed in tandem to screen for new antimicrobials. The consequence of this is that these environments are not found individually in vivo. In reality, these distinct phenotypes fuse to form a third phenotype, the clinical phenotype (Figure 1). It is this clinical phenotype that requires future in vitro modelling if novel drug screening is to be met with any success.
